# Re-establishing the utility of tetracycline-class antibiotics for current challenges with antibiotic resistance

**DOI:** 10.1080/07853890.2022.2085881

**Published:** 2022-06-20

**Authors:** Kerry L. LaPlante, Abhay Dhand, Kelly Wright, Melanie Lauterio

**Affiliations:** a College of Pharmacy, University of Rhode Island, Kingston, RI, USA; b Westchester Medical Center, New York Medical College, Valhalla, NY, USA; c Paratek Pharmaceuticals, Inc, King of Prussia, PA, USA

**Keywords:** Tetracycline, omadacycline, eravacycline, tigecycline, efficacy, safety, antibiotic resistance, *Clostridioides difficile* infection, antibiotics, penicillin allergy

## Abstract

The progressive increase in antibiotic resistance in recent decades calls for urgent development of new antibiotics and antibiotic stewardship programs to help select appropriate treatments with the goal of minimising further emergence of resistance and to optimise clinical outcomes. Three new tetracycline-class antibiotics, eravacycline, omadacycline, and tigecycline, have been approved within the past 15 years, and represent a new era in the use of tetracyclines. These drugs overcome the two main mechanisms of acquired tetracycline-class resistance and exhibit a broad spectrum of *in vitro* activity against gram-positive, gram-negative, anaerobic, and atypical pathogens, including many drug-resistant strains. We provide an overview of the three generations of tetracycline-class drugs, focussing on the efficacy, safety, and clinical utility of these three new third-generation tetracycline-class drugs. We also consider various scenarios of unmet clinical needs where patients might benefit from re-engagement with tetracycline-class antibiotics including outpatient treatment options, patients with known β-lactam antibiotic allergy, reducing the risk of *Clostridioides difficile* infection, and their potential as monotherapy in polymicrobial infections while minimising the risk of any potential drug-drug interaction.

**KEY MESSAGES**
- The long-standing safety profile and broad spectrum of activity of tetracycline-class antibiotics made them a popular choice for treatment of various bacterial infections; unfortunately, antimicrobial resistance has limited the utility of the early-generation tetracycline agents.
- The latest generation of tetracycline-class antibiotics, including eravacycline, tigecycline, and omadacycline, overcomes the most common acquired tetracycline resistance mechanisms.
- Based on *in vitro* characteristics and clinical data, these newer tetracycline agents provide an effective antibiotic option in the treatment of approved indications in patients with unmet clinical needs – including patients with severe penicillin allergy, with renal or hepatic insufficiency, recent *Clostridioides difficile* infection, or polymicrobial infections, and those at risk of drug–drug interactions.

## Introduction

Over the past decade, there has been a substantial increase in the number of infections caused by bacteria that are resistant to one or more antibiotics [1]. Annually, more than 2.8 million antibiotic-resistant infections occur in the United States with approximately 35,000 deaths attributed to antibiotic-resistant infections [1]. This increased morbidity and mortality caused by antibiotic-resistant infections is associated with a considerable economic burden: $4.6 billion is spent annually to treat infections caused by six multidrug-resistant bacteria alone [2]. The US Centres for Disease Control and Prevention (CDC) has identified major bacterial and fungal drug-resistance threats, of which carbapenem-resistant *Acinetobacter* and Enterobacterales, drug-resistant *Neisseria gonorrhoeae*, and *Clostridioides difficile* pose urgent threats [1]. Recognising the need for creative approaches to address the problem of a dwindling antimicrobial research and development pipeline, in 2010 the Infectious Diseases Society of America launched the 10 × ’20 initiative to encourage the development of 10 new systemic antibacterial agents by 2020 [3]. In total, 14 new agents were approved between 2010 and 2020, with several others in late-stage clinical development [4]. Of these newly approved agents, two were novel third-generation tetracycline-class antibiotics (eravacycline and omadacycline), with a third (tigecycline) approved by the US Food and Drug Administration (FDA) in 2005. This commentary focuses on the history of the three generations of tetracycline-class antibiotics, their mechanism of action, the emergence of resistance, and relevant data regarding the clinical utility of newer tetracycline agents.

## History and clinical utility of tetracycline-class drugs

The first tetracycline-class drugs were discovered in the late 1940s, isolated from *Streptomyces* spp [5]. Aureomycin (also known as chlortetracycline) was approved for use in the United States in 1948; the other three commonly used first- and second-generation tetracycline-class drugs (tetracycline, doxycycline, and minocycline) were approved in 1954, 1967, and 1971, respectively. No new tetracycline-class drugs were then developed until the third generation in the 2000s [5].

Tetracycline-class drugs inhibit bacterial protein synthesis by binding to bacterial ribosomes and interacting with the highly conserved 16S ribosomal RNA (rRNA) in the 30S ribosomal subunit [6]. The drug class demonstrates a broad spectrum of activity against a wide range of gram-positive, gram-negative, and atypical pathogens, resulting in the extensive use of the tetracycline class in both humans and animals after the drugs were initially discovered [5]. Indications for treatment of bacterial infections include pneumonia; skin infections; bone and joint infections; sexually transmitted infections including chlamydia, syphilis, and gonorrhoea; intra-abdominal infections; biothreat pathogens, including *Yersinia pestis, Bacillus anthracis*, and *Francisella tularensis*; and other specific bacterial pathogens such as *Rickettsia* spp, *Borrelia* spp, and nontuberculous mycobacteria. Tetracycline-class agents are recommended as first-line treatment options for many of these indications [7–13].

## Tetracycline resistance

While tetracycline-class drugs have a broad spectrum of antibacterial activity and many clinical applications, their utility has declined over time through the emergence of antibiotic resistance. In the early antibiotic era (up until the mid-1950s), most commensal and pathogenic bacteria remained susceptible to tetracyclines; however, within 2  years of the approval of aureomycin, resistance had emerged in *Staphylococcus aureus* [14]. In 1954, resistant strains of *Streptococcus pyogenes* were first noted in patients with burn injuries [15]. The first-generation tetracycline and second-generation minocycline and doxycycline demonstrated improved activity versus aureomycin against *S. aureus*, yet many *Streptococcus* species remained resistant. By 1968, approximately 23% of *Streptococcus pneumoniae* isolates were resistant to all three drugs, and at least half of these resistant infections were acquired outside of hospital settings [16]. The increasing rates of acquired resistance to tetracycline-class drugs during the 1960s and 1970s, and the growing availability of other antibiotic agents such as cephalosporins and fluoroquinolones [17], led to this class being used mainly as a second-line treatment option.

**Figure 1.**
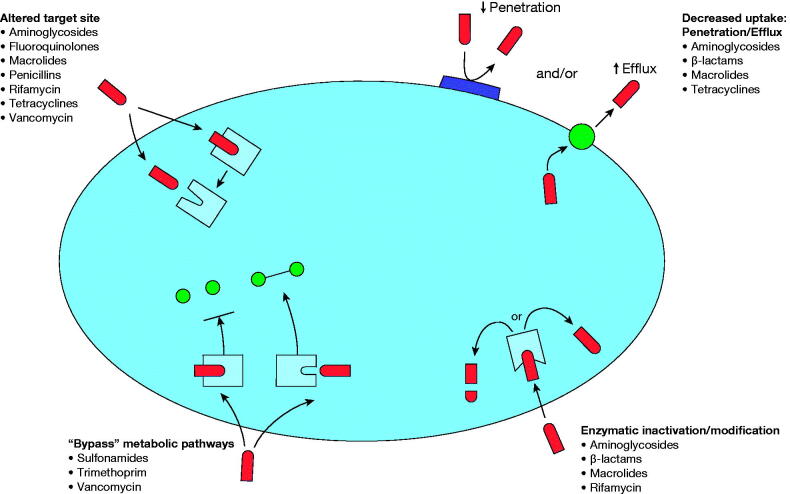
Overview of the four major mechanisms of antibiotic resistance, and antibiotic classes affected by each mechanism. Adapted with permission from Hawkey [106].

Multiple mechanisms confer acquired resistance to the tetracycline class, the most common being efflux pumps and ribosomal protection proteins (Figure 1). Efflux pumps are antiporters that exchange a monocationic magnesium–tetracycline complex for a proton, thus actively pumping antibiotics out of the bacterial cell. Efflux pumps are found in both gram-positive, e.g. Tet(K) and Tet(L), and gram-negative bacteria, e.g. Tet(A) and Tet(B), including *Staphylococcus*, *Streptococcus*, *Klebsiella*, and *Escherichia* species [5,18,19]. Ribosomal protection proteins such as Tet(O) and Tet(M), found in both gram-positive and gram-negative organisms, are cytoplasmic proteins that protect ribosomes from the inhibitory action of tetracycline-class drugs [19]. Although the exact mechanism of resistance is unclear, it is likely that the proteins cause a conformational change in the structure of the ribosome, either preventing tetracycline binding or resulting in dissociation [18]. Two other acquired tetracycline resistance mechanisms that are less common include target modification, where a mutation in the ribosomal RNA of the target binding site reduces antibiotic binding affinity; and drug degradation, where enzymatic action mediated by *tet*(X) genes degrades antibiotic products [5,18]. However, *tet*(X) could be an emerging threat to susceptibility to all tetracyclines particularly gram-negative pathogens [20]. In addition to these acquired resistance mechanisms, intrinsic resistance to tetracycline-class drugs has been noted in *Providencia* spp, *Proteus mirabilis*, and *Pseudomonas aeruginosa* among gram-negative organisms [21–23].

Since the 1990s, the prevalence of *S. aureus*, *S. pneumoniae*, and *S. pyogenes* resistance to β-lactams and non-β-lactams, such as macrolides and tetracycline-class antibiotics, has steadily increased. The SENTRY Antimicrobial Surveillance Program reported that the global occurrence of methicillin-resistant *S. aureus* (MRSA) increased from 33% in 1997–2000 to 44% in 2005–2008, with the highest rates in North America [24]. A recent analysis of data from 329 US hospitals also showed that *S. pneumoniae* macrolide resistance was >25% in most regions and nearly 40% overall isolates tested [25]. In general, similar rates of resistance are seen in *Streptococcus* spp to the first-generation tetracycline and second-generation doxycycline, whereas *S. aureus* resistance rates decrease with later-generation tetracyclines [26–28]. As rates of antimicrobial resistance continue to increase, new antibiotics, including third-generation tetracycline-class drugs, offer an important therapeutic option for various bacterial infections.

## Generations of tetracycline-class drugs: similarities and differences

**Figure 2.**
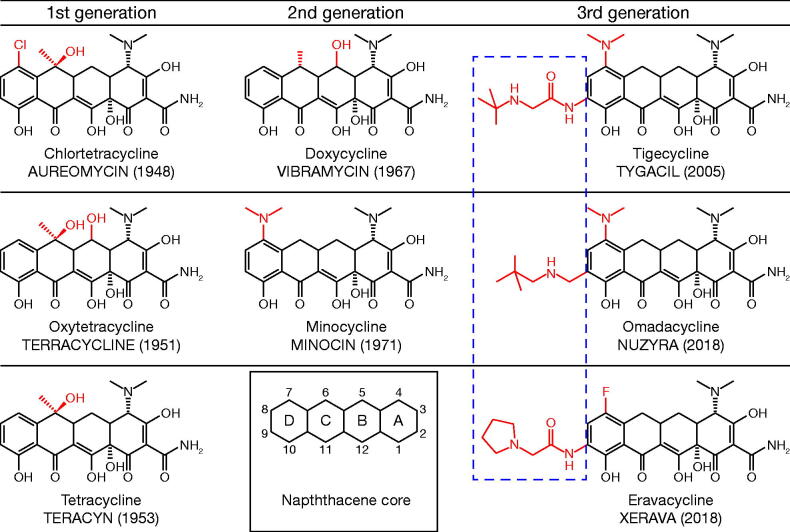
Chemical structures of tetracyclines. Chemical structures of (A–C) rst generation tetracyclines. (A) chlortetracycline (aureomycin), (B) oxytetracycline (terracycline) and (C) tetracycline (teracyn), (D–E) second generation tetracyclines; (D) doxycycline (vibramycin) and (E) minocycline (minocin), and (F–G) third generation tetracyclines; (F) the glycylcycline tigecycline (tygacil), (G) the aminomethylcyclineomadacycline (PTK 0796) and (H) the uorocycline eravacycline (TP–434). The numbers in parentheses indicates the year the antibiotic was discovered/reported. The inset of the DCBA naphthacene core provides the carbon atom assignments for rings A–D.

All tetracycline-class drugs share a core chemical structure, with variations in the side groups (Figure 2). The generations of tetracycline-class drugs are defined by the methodology used to develop the drug: the first generation is obtained from biosynthesis, the second generation is semi-synthetic products, and the third generation is entirely synthetic and can therefore have more elaborate side chains than earlier generations; this enhances their antibacterial activity (Figure 2). In all tetracycline-class drugs, the amino group present in the C4 position of the A ring is vital for antibiotic activity [29]. While the second-generation drugs have increased oral bioavailability compared with the first generation, they largely did not overcome the acquired resistance mechanisms through efflux pumps and ribosomal protection proteins, with the exception of *S. aureus* efflux pumps for minocycline [30]. Additionally, inducible resistance against doxycycline has been observed in previously susceptible community-associated MRSA isolates [31]. In the third-generation tetracycline-class drugs, the addition of a lipophilic side group at the C9 position on the D ring enhances the antibiotic activity further, and all three are designed to overcome the two main tetracycline resistance mechanisms [27]. While only tetracycline is still in routine use from the first generation of tetracycline-class drugs (developed between 1948 and 1963), two second-generation drugs (developed between 1965 and 1972), doxycycline and minocycline, are also still routinely used in humans. Tetracycline is currently only available in an oral formulation, whereas second-generation drugs are available in both oral and intravenous (IV) formulations. Of the third-generation drugs, eravacycline and tigecycline are available only in IV formulation, whereas omadacycline is available in both oral and IV formulations.

## *In vitro* activity of tetracycline-class antibiotics

Tetracycline-class drugs show a broad range of *in vitro* activity against gram-positive, gram-negative, anaerobic and atypical pathogens [5]. While the clinical relevance of *in vitro* activity is unknown, third-generation tetracycline-class drugs have improved activity against many common community-acquired bacterial pneumonia (CABP) and skin and skin structure infection pathogens when compared with first-generation tetracycline (Table 1) [32].

**Table 1.** *In vitro* activity of tetracycline and third-generation tetracycline-class drugs against select gram-positive, gram-negative, and anaerobic pathogens [35,103,104].

Organism (*n*)	Tetracycline		Tigecycline		Omadacycline		Eravacycline	
	MIC_90_ (mg/L)	%S*	MIC_90_ (mg/L)	%S*	MIC_90_ (mg/L)	%S*	MIC_90_ (mg/L)	%S*
*Bacteroides fragilis*	–^a^	–^a^	4	–^a^	–^a^	–^a^	1	–^a^
*Enterococcus faecalis*	>16	24.8	0.12	99.1	0.25	98.2	0.06	94.5
Vancomycin resistant	>16	17.0	0.12	100	0.12	–^a^	0.12	89.8
*Escherichia coli*	>16	69.3	0.25	100	2	–^a^	0.25	99.2
ESBL phenotype	>8	88.1	0.25	100	2	–^a^	–^a^	–^a^
*Haemophilus influenzae*	1	99.7	0.25	90.5	1	99.7	0.25	–^a^
*Klebsiella pneumoniae*	>16	77.0	1	96.8	4	91.0	1	85.7
ESBL phenotype	>16	39.8	2	92.0	16	73.9	–^a^	–^a^
*Staphylococcus aureus*	0.25	98.3	0.12	100	0.25	97.8	0.12	84.5
Methicillin resistant	0.12	99.7	0.25	95.2	0.5	97.2	0.12	80.8
*Streptococcus pneumoniae*	>4^a^	79.7^a^	0.12	86.9	0.12	97.6	0.015	^a^
Penicillin resistant, oral	>4^a^	48.5^a^	0.12	84.8	0.12	93.9	0.016	–^a^
Macrolide resistant	>4^a^	61.0^a^	0.12	84.9	0.12	95.1	0.016	–^a^
Tetracycline resistant	>4^a^	0	0.12	82.4	0.12	91.2	0.016	–^a^
*Streptococcus pyogenes*	>4	73.9	0.12	98.6	0.12	–^a^	0.03	100

* Susceptibility data are derived from FDA identified breakpoints. Criteria as published by CLSI.

CLSI: Clinical and Laboratory Standards Institute; ESBL: extended-spectrum β-lactamase; FDA: Food and Drug Administration: MIC: minimum inhibitory concentration.

^a^ Breakpoints unavailable.

Minimum inhibitory concentrations (MICs) obtained from *in vitro* studies provide the key context for antibiotic selection against those pathogens. Lower MICs indicate that less drug is required for inhibition of organism growth, thus providing greater antimicrobial activity than a drug with a higher MIC value, assuming pharmacodynamic (PD) exposure is the same [33]. Susceptibility testing varies and is based on breakpoint criteria developed by two organisations, the Clinical and Laboratory Standards Institute (CLSI) and the European Committee on Antimicrobial Susceptibility Testing (EUCAST) [27,28]. In general, CLSI breakpoints have been approximately fourfold higher than EUCAST, but CLSI breakpoints for *S. pneumoniae* were revised in 2013 to more accurately reflect the presence or absence of *tet* resistance genes [27]. There remains an unmet need for harmonisation of tetracycline breakpoints against other pathogens, as variations between the two criteria show large ranges in susceptibility rates against other pathogens, such as MRSA (Table 2).

**Table 2.** Susceptibility rates for first- and second-generation tetracycline-class drugs against common gram-positive pathogens [27,28].

Organism	Antibiotic^a^	MIC (µg/mL)		% Susceptible / % Resistant	
		50%	90%	CLSI	EUCAST
*Streptococcus pneumoniae*	Tetracycline	0.5	>8	73.2 / 26.7	73.2 / 26.7
	Doxycycline	0.25	8	71.3 / 26.8	73.9 / 24.7
	Minocycline	NR	NR	71.7 / 27.3^b^	72.7 / 26.4
*Streptococcus pyogenes*	Tetracycline	≤0.25	>8	80.3 / 19.7	79.6 / 19.7
	Doxycycline	0.12	8	81.2 / 16.0	80.2 / 18.8
	Minocycline	NR	NR	78.0 / 20.7	78.0 / 22.0
Methicillin-resistant *Staphylococcus aureus*	Tetracycline	≤0.25	2	91.2 / 8.0	88.1 / 9.0
	Doxycycline	0.12	1	96.2 / 0.6	93.5 / 5.5
	Minocycline	NR	NR	97.2 / <0.1	88.3 / 11.3

CLSI: Clinical and Laboratory Standards Institute; EUCAST: European Committee on Antimicrobial Susceptibility Testing; MIC: minimum inhibitory concentration; NR: not recorded.

^a^Tetracycline is considered a first-generation tetracycline-class drug. Doxycycline and minocycline are second-generation tetracycline-class drugs.

^b^Using CSLI 2013 susceptibility breakpoint for doxycycline of ≤0.25 μg/mL applied to the minocycline.

For the tetracycline-class agents, MICs for a particular pathogen are generally substantially lower for the third-generation drugs relative to values for tetracycline and doxycycline [34]. Third-generation tetracycline-class drugs also maintain their activity against pathogens that are resistant to first- and second-generation drugs, and overall, show more potent antibacterial activity than older generations (Table 3) [32,34–37]. A full list of susceptible pathogens is provided in Table 4.

**Table 3.** *In vitro* activity of tetracycline-class drugs in the presence and absence of acquired tetracycline resistance genes [34,98,102,103].

Strain	Tet^R^ determinant	Mechanism type	MIC range (µg/mL)				
			Firstgeneration	Secondgeneration	Third-generation		
			Tetracycline^a^	Doxycycline^a^	Omadacycline^a^	Eravacycline^b^	Tigecycline^b^
*Staphylococcus aureus*	None	–	≤0.06–0.25	≤0.06–0.125	≤0.06–0.5	0.015–0.12	0.03–0.25
	*tet*(M)	RPPs	32 to >64	2–16	0.125–1	NR	NR
	*tet*(K)	Efflux pump	16–32	1–4	0.125–0.25	0.063	0.13
*Streptococcus pneumoniae*	None	–	≤0.06–0.25	≤0.06–0.25	≤0.06–0.25	0.004–0.03	0.015–0.12
	*tet*(M)	RPPs	4–64	2–4	≤0.06	0.016	≤0.016
β-hemolytic streptococci^c^	None	–	≤0.06–0.125	≤0.06	≤0.06–0.50	0.004–0.25	≤0.008–0.25
	*tet*(M)	RPPs	4–64	2–16	≤0.06–0.50	NR	NR
	*tet*(O)	RPPs	32–64	8	≤0.06–0.25	NR	NR

MIC: minimum inhibitory concentration; NR: not recorded; RPP: ribosomal protection protein.

^a^ Data for first- and second-generation tetracycline-class drugs and omadacycline adapted from [34].

^b^ Data for eravacycline and tigecycline adapted from [98,104,105].

^c^ *S. pyogenes and S. agalactiae*.

**Table 4.** Indications and susceptible pathogens for third-generation tetracycline-class drugs [9,10,12].

Antibiotic	Indication	Susceptible microorganisms
Tigecycline	Complicated skin and skin structure infection	*Escherichia coli*, *Enterococcus faecalis* (vancomycin-susceptible isolates), *Staphylococcus aureus* (methicillin-susceptible and -resistant isolates), *Streptococcus agalactiae*, *Streptococcus anginosus* group (includes *S*. *anginosus, S. intermedius*, and *S. constellatus*), *Streptococcus pyogenes*, *Enterobacter cloacae*, *Klebsiella pneumoniae*, and *Bacteroides fragilis*
	Community-acquired bacterial pneumonia	*Streptococcus pneumoniae* (penicillin-susceptible isolates), *Haemophilus influenzae* (β-lactamase negative isolates), and *Legionella pneumophila*
	Complicated intra-abdominal infection	*Citrobacter freundii*, *E. cloacae*, *E. coli*, *Klebsiella oxytoca*, *K. pneumoniae*, *E. faecalis* (vancomycin-susceptible isolates), *S. aureus* (methicillin-susceptible and -resistant isolates), *S. anginosus* group, *B. fragilis*,* Bacteroides thetaiotaomicron*, *Bacteroides uniformis*, *Bacteroides vulgatus*, *Clostridium perfringens*, and *Peptostreptococcus micros*
Eravacycline	Complicated intra-abdominal infection	*E. coli*, *K. pneumoniae*, *C. freundii*, *E. cloacae*, *K. oxytoca*, *E. faecalis*, *Enterococcus faecium*, *S. aureus*, *S. anginosus* group, *C. perfringens*, *Bacteroides* spp, and *Parabacteroides distasonis*
Omadacycline	Acute bacterial skin and skin structure infection (ABSSSI)	*S. aureus* (methicillin-susceptible and -resistant isolates), *Staphylococcus lugdunensis*, *S. pyogenes*, *S. anginosus* group, *E. faecalis*, *E. cloacae*, and *K. pneumoniae*
	Community-acquired bacterial pneumonia	*S. pneumoniae*, *S. aureus (*methicillin-susceptible isolates), *H. influenzae*, *Haemophilus parainfluenzae*, *K. pneumoniae*, *L. pneumophila*, *Mycoplasma pneumoniae*, and *Chlamydophila pneumoniae*

Third-generation tetracycline-class agents evade the two most common mechanisms of tetracycline resistance, but the emergence of resistance to these newer drugs remains a concern. Current resistance mechanisms to tigecycline mostly involve overexpression of efflux pumps and enzymatic inactivation, including by *tet*(X), a gene encoding a flavin-dependent monooxygenase [38–40]. Resistance to all tetracycline-class antibiotics has now been noted in carbapenem- and colistin-resistant multidrug-resistant pathogens that carry the *tet*(X) gene or homologs [41,42]. A global survey of the *in vitro* activity of tigecycline and comparators assessing trends in susceptibility from 2004 to 2013 indicated that susceptibility remained high, although decreases in susceptibility over time had been noted, for example against *Enterobacter* spp in Latin America [43].

### Pharmacokinetics/pharmacodynamics of tetracycline-class drugs

Pharmacokinetic (PK)/PD characteristics, and thus drug dosage and dosing frequency, and available formulations of the tetracycline-class agents, vary: overall, tetracycline-class drugs have a longer half-life than older drugs, which supports once- or twice-daily administration (Table 5). Eravacycline and tigecycline, despite their prolonged half-lives, are dosed twice daily to improve tolerability related to concentration-dependent gastrointestinal adverse events (AEs) [44–46]. Dose adjustment is not required for the newer generation of tetracycline agents in patients with renal impairment. Doses need to be adjusted only for eravacycline and tigecycline in patients with severe hepatic impairment [8,10,12].

**Table 5.** Pharmacokinetic parameters of tetracycline-class drugs [8–13,47].

Antibiotic	Maintenance dose	Dosing frequency	Food effect (*C*_max_ decrease)	Protein binding	Half-life	Metabolism	Excretion
Tetracycline	Oral: 250/500 mg	Oral: BID to QID	50%	55–64%	6–11 h	Yes	Fecal: 20–60% Renal: 30%
Doxycycline	IV: 100 mg Oral: 100 mg	Oral: QD or BID IV: BID	20%		12–25 h	Yes	Renal: 40%
Minocycline	IV: 100 mg Oral: 50/75/100 mg	Oral: BID	–	76%	11–24 h	Yes	Fecal: 20–35% Renal: 5–12%
Omadacycline	IV: 100 mg Oral: 300 mg	Oral: QD IV: QD	40–59%	20%	16 h	Yes	Fecal (oral): 81% Renal (IV/oral): 27%/14%
Tigecycline	IV: 50 mg	IV: BID	NA	71–89%	42 h	None	Biliary/Fecal: 59% Renal: 33%
Eravacycline	IV: 1 mg/kg	IV: BID	NA	79–90%	20 h	CYP3A4- and FMO-mediated oxidation	Biliary/Fecal: 47% Renal: 34%

BID: twice daily; IV: intravenous; NA: not applicable; QD: daily; QID: four times daily.

#### Pharmacokinetics

**Figure 3.**
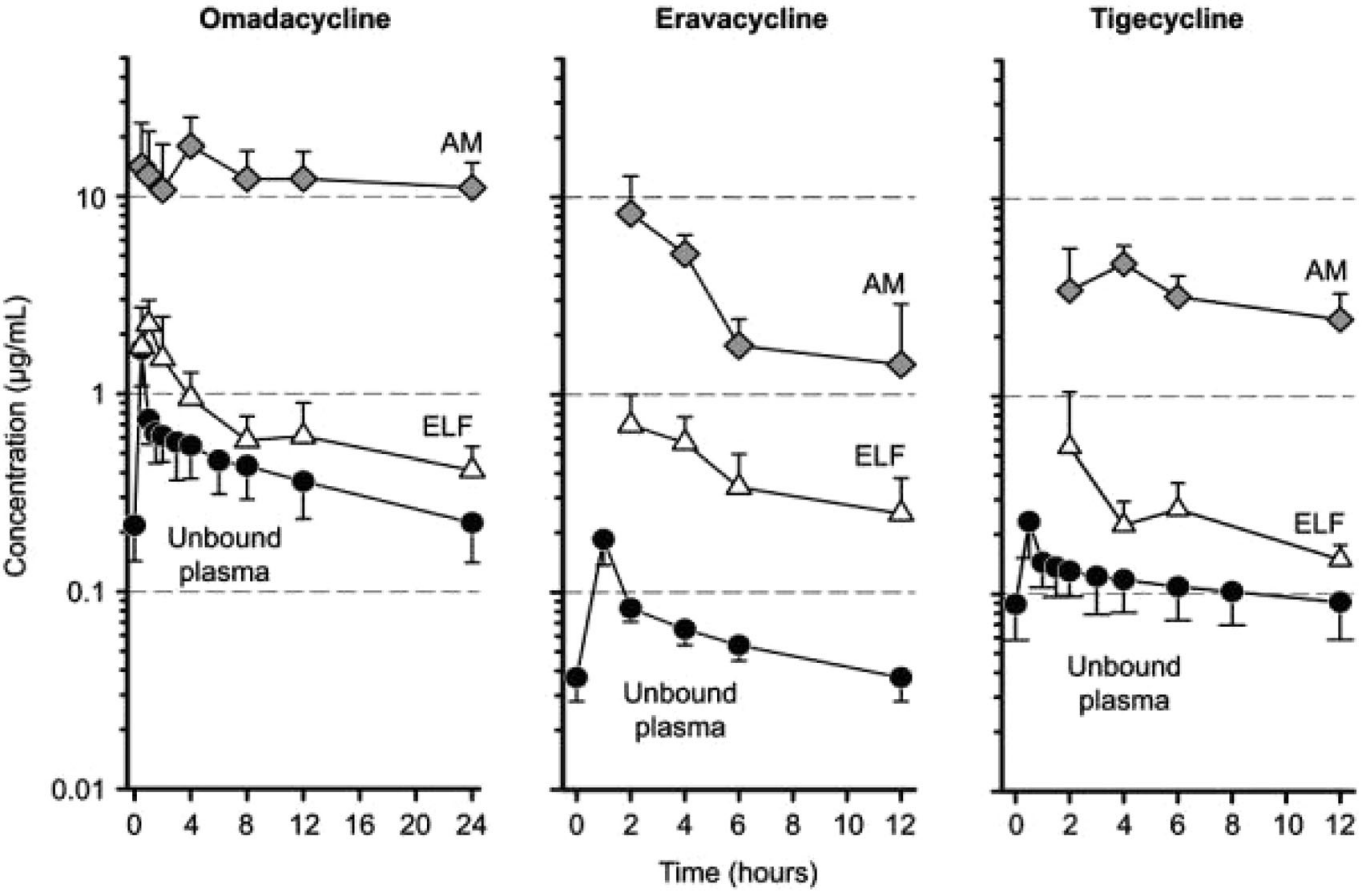
Mean (standard deviation) unbound plasma, alveolar macrophages (AM), and epithelial lining fluid (ELF) concentration–time curves after multiple intravenous doses in healthy patients. Taken from [48]. Originally published by and used with permission from Dove Medical Press Ltd.

Absorption of tetracycline-class drugs is variable and ranges from approximately 25–60% for first-generation drugs to (near) complete absorption for later generations [47]. The absorption of all tetracycline-class drugs is impaired by antacids containing aluminium, calcium, or magnesium, bismuth subsalicylate, and iron-containing preparations [47]. Data on first- and second-generation drugs are limited (owing to their development and approval before current regulatory standards were in place), but third-generation tetracycline-class antibiotics are known to have a high volume of tissue distribution including in pulmonary tissues [47,48]. Omadacycline, eravacycline, and tigecycline concentrations in unbound plasma, alveolar macrophages, and epithelial lining fluid are high, with a greater magnitude of omadacycline concentrations than those for the other two drugs (Figure 3). Tetracycline-class antibiotics are all metabolised, with the exception of tigecycline (Table 5) [47]. Excretion varies across the tetracycline class, but most of the drugs have a combination of majority biliary/fecal and some renal excretion [47].

#### Pharmacodynamics

The bacterial killing effect of second- and third-generation tetracycline agents has been evaluated in various *in vitro* and *in vivo* studies. Doxycycline demonstrates time-dependent killing at 2–4 times the MIC (i.e. low serum concentrations) but dose-dependent killing at higher concentrations (8–16 times the MIC) [49]. Post-antibiotic effect (PAE), the period after removal of the antibiotic after which no further bacterial growth occurs, is similar to doxycycline against both gram-negative and gram-positive organisms and is concentration dependent [49]. PAE for omadacycline varies with the pathogen tested, with similar PAE for tigecycline and eravacycline against *S. aureus*, *S. pneumoniae*, and *E. coli* [50,51].

As tetracycline-class drugs have substantial post-antibiotic effects, the area under the free concentration-time curve (*f*AUC) to MIC ratio is an important predictor of *in vivo* activity [46,47]. *In vitro* and *in vivo* studies of doxycycline showed *f*AUC/MICs of 24 and 25 to be associated with net stasis against *S. pneumoniae* and S*. aureus*, respectively [46,52]. For eravacycline, *f*AUC/MICs of 28.0 and 32.6 have been associated with net stasis and 1_log_-kill endpoints, respectively, against *E. coli* in murine thigh infection models [46]. In PD studies of omadacycline, mean *f*AUC/MICs of 25.7 and 23.0 were required for stasis against *S. pneumoniae* and *E. coli*, respectively [53]. Data from neutropenic murine thigh and lung studies indicate the median *f*AUC/MICs required to achieve stasis for *S. aureus* and 1-log_10_ reduction for *S. pneumoniae* are 21.9 and 17.4, respectively [54,55]. For tigecycline, the mean effective exposure index at 80% for *f*AUC/MIC was 5.4 µg/mL (range, 2.8–13 µg/mL) against *S. aureus* in a murine thigh model [56].

## Clinical data on third-generation tetracycline-class antibiotics

Third-generation tetracycline-class agents are approved for multiple therapeutic indications, including skin and skin structure infections, CABP, and complicated intra-abdominal infection (cIAI) (Table 4). Approval for these indications was based on efficacy and safety data from phase 3 studies using standard-of-care oxazolidinones or glycopeptides, fluoroquinolones, or carbapenems as respective comparators [10,12,57]. These studies were all designed to align with then-current FDA guidance, which leads to some of the differences in endpoints as reported below.

### Efficacy data

#### Community-acquired bacterial pneumonia

Omadacycline and tigecycline are approved for the treatment of adults with CABP. In the phase 3 OPTIC study, omadacycline was non-inferior to moxifloxacin in the treatment of hospitalised adults with CABP, based on the primary outcome of early clinical response (ECR), defined as survival with the improvement of at least two of four symptoms (cough, sputum production, pleuritic chest pain, and dyspnea) and no worsening of symptoms at 72–120 h, without receipt of rescue antibacterial therapy [58]. Similar findings were observed at post-treatment evaluation (PTE; 5–10 days after last dose of the study drug). Tigecycline also demonstrated non-inferiority to levofloxacin in adults hospitalised for CABP based on clinical cure (all signs and symptoms of pneumonia improved or resolved, with no worsening of symptoms or new signs, and no requirement for further antibiotic therapy) at 10–21 days post therapy [59–62].

#### Skin and skin structure infections

Omadacycline and tigecycline are also indicated for the treatment of skin and skin structure infections; omadacycline is FDA approved for the treatment of acute bacterial skin and skin structure infections (ABSSSIs), whereas tigecycline is approved for the treatment of complicated skin and skin structure infections (cSSSIs). ABSSSI is considered a more specific categorisation and is defined as a bacterial infection of the skin with a lesion size area of ≥75 cm^2^, whereas cSSSIs involve abnormal skin, wounds, or infections in immunocompromised individuals or infections requiring surgery [62]. In the phase 3 OASIS-1 and OASIS-2 studies in adult patients with ABSSSIs, IV-to-oral or oral-only omadacycline was non-inferior to linezolid at ECR (48–72 h after the first dose), based on a > 20% reduction in lesion size, with similar rates of clinical success observed at PTE [63,64]. Tigecycline achieved non-inferiority to vancomycin and aztreonam at the test-of-cure visit (12–92 days after the last dose of the study drug) based on the resolution of signs and symptoms such that no further antibiotic therapy was required [65,66].

#### Complicated intra-abdominal infections

Tigecycline and eravacycline are approved by the FDA for the treatment of complicated IAIs. In the phase 3 IGNITE1 and IGNITE4 studies, eravacycline demonstrated non-inferiority to ertapenem and meropenem, respectively, in patients with cIAI based on clinical efficacy 25–31 days after the first dose of the study drug [67,68]. Clinical efficacy was defined as complete resolution or significant improvement of signs or symptoms of the index infection such that no additional antibacterial therapy, surgical, or radiological intervention was required. Similarly, tigecycline demonstrated non-inferiority to imipenem–cilastatin for the treatment of cIAI, based on clinical cure 12–42 days after the first dose of the study drug [69,70]. In these studies, the cure was defined as resolution of the IAI process following the course of study drug and initial intervention.

### Safety, tolerability, and drug interactions

#### Tetracycline class effects

All tetracycline-class drugs, including the third-generation agents, come with warnings and precautions including tooth discolouration and enamel hypoplasia during tooth development (last half of pregnancy up until 8 years of age), and inhibition of bone growth (second trimester of pregnancy until 8 years of age). Tigecycline also has a boxed warning for increased mortality risk and should be reserved for situations when alternative treatments are not suitable [10]. Other tetracycline-class AEs include photosensitivity, pseudotumor cerebri, and anti-anabolic action. As seen with other drug classes, while these AEs are attributed to the tetracycline class as a whole, the magnitude of the effect varies between specific drugs within the class (e.g. photosensitivity [71]). Tetracycline-class drugs have been shown to depress plasma prothrombin activity; patients taking anticoagulants may therefore need to be monitored or have their oral anticoagulant dosage adjusted while taking tetracycline-class drugs [9,10,12]. There is impaired absorption of oral tetracycline-class drugs by antacids containing aluminium, calcium, or magnesium, bismuth subsalicylate, and iron-containing preparations [9].

#### C. difficile infection

The potential for *C. difficile*-associated diarrhoea and the associated label warning is common to all antibiotics, although there is evidence of lower rates of *C. difficile* infection (CDI) with the tetracycline class than with other classes of antibiotics, such as fluoroquinolones and third-generation cephalosporins [72–75]. Tigecycline has been used in select cases, usually for severe disease, as a successful adjunctive treatment for CDI [76]. Across the phase 3 studies of omadacycline, no cases of CDI were observed in the omadacycline treatment groups [77]. A retrospective study of 50 patients receiving eravacycline as an inpatient or outpatient parenteral antibiotic therapy reported only one case of post-treatment CDI, where 40% of these patients had experienced CDI in the 12 months before receiving eravacycline [78]. While the mechanism of decreased risk of CDI with tetracycline-class drugs remains unclear, high drug concentrations in the bowel, less disruption to the gut microbiota than observed with other antibiotics, and *in vitro* and *in vivo* activity against *C. difficile* may all play a role [75,79,80].

#### Tigecycline

Pooled data across various studies show that nausea and vomiting were the most frequently reported AEs (Table 6). These gastrointestinal AEs are *C*_max_ related and thus dose-limiting; in general, the maximum single dose is 100 mg IV [44]. However, higher doses have been used in an attempt to maximise efficacy, which resulted in increased AEs and discontinuation [81], leading to attempts at reduced dosing of 25 mg once or twice daily in some long-term utilisation studies to minimise gastrointestinal side effects (e.g. [82]). Across clinical trials, 7% of 2514 patients discontinued tigecycline due to AEs, compared with 6% of those taking the comparator drugs [10].

**Table 6.** Most frequently (≥2%) occurring adverse events from pooled phase 3 clinical studies of third-generation tetracycline-class drugs [10,12,77].

Indication	Adverse event	Tetracycline-class agent	Comparator
*Omadacycline*			
ABSSSI, CABP		*N* = 1073	*N* = 1077^a^
	Nausea	15%	8%
	Vomiting	8%	3%
	ALT increased	4%	4%
	AST increased	3%	4%
	Headache	3%	2%
	Infusion-site extravasation	3%	2%
	Wound infection	3%	2%
	Cellulitis	3%	2%
	Diarrhoea	2%	5%
	Subcutaneous abscess	2%	3%
	Hypertension	2%	1%
*Tigecycline*			
CABP, cSSSI, cIAI		*N* = 2514	*N* = 2307^b^
	Nausea	26%	13%
	Vomiting	18%	9%
	Diarrhoea	12%	11%
	Infection	7%	5%
	Abdominal pain	6%	4%
	Headache	6%	7%
	SGPT increased	5%	5%
	Anemia	5%	6%
	Hypoproteinemia	5%	3%
	SGOT increased	4%	5%
	Phlebitis	3%	4%
	Rash	3%	4%
	Alkaline phosphatase increased	3%	3%
	Dizziness	3%	3%
	Asthenia	3%	2%
	Amylase increased	3%	2%
	Abnormal healing	3%	2%
	BUN increased	3%	1%
	Abscess	2%	2%
	Dyspepsia	2%	2%
	Pneumonia	2%	2%
	Bilirubinemia	2%	1%
	Hyponatremia	2%	1%
*Eravacycline*			
cIAI		*N* = 520	*N* = 517^c^
	Infusion-site reactions	8%	2%
	Nausea	7%	0.6%
	Vomiting	4%	3%
	Diarrhoea	2%	2%

^a^ Comparators: linezolid, moxifloxacin.

^b^ Comparators: vancomycin–aztreonam, imipenem–cilastatin, levofloxacin, linezolid.

^c^ Comparators: ertapenem, meropenem.

ABSSSI: acute bacterial skin and skin structure infection; ALT: alanine aminotransferase; AST: aspartate aminotransferase; BUN: blood urea nitrogen; CABP: community-acquired bacterial pneumonia; cIAI: complicated intra-abdominal infection; cSSSI: complication skin and skin structure infection; SGOT: serum glutamic oxaloacetic transaminase; SGPT: serum glutamic pyruvic transaminase.

A meta-analysis of phase 3 and 4 clinical trials demonstrated an increase in all-cause mortality in tigecycline-treated patients compared with controls, with a risk difference of 0.6% (95% CI 0.1, 1.2). The cause of this increase has not been established, and the increase was also seen when limited to approved indications. The greatest differences in mortality versus controls were observed in patients with ventilator-assisted pneumonia [10], which is not an FDA-approved indication for tigecycline.

#### Eravacycline

Nausea and vomiting are also frequently reported AEs for eravacycline (Table 6), with a trend towards increasing incidence of nausea and vomiting with increasing eravacycline *C*_max_ [46]. Rates of nausea were higher with twice- versus once-daily administration (11% vs 2%), but rates of vomiting were lower (2% vs 6%) [83]. Local infusion-site reactions, most of which were mild, were also more frequent with eravacycline than the comparator drug in phase 3 studies [67,68]. Across clinical trials, 2% of 520 patients receiving eravacycline discontinued due to AEs, compared with 2% of patients who received the comparator [12]. Mortality rates were similar to those observed for the comparator drugs [67,68].

#### Omadacycline

Nausea and vomiting were the most frequently observed AEs in the omadacycline group (15% and 8%, respectively) (Table 6). This was largely driven by higher rates of nausea and vomiting reported during the high dose oral-only (450 mg) loading period in the first 2 days of the OASIS-2 study [63,77], with rates similar to the comparators in the IV-to-oral OPTIC (omadacycline, 2.4% and 2.6%; moxifloxacin, 5.4% and 1.5% for nausea and vomiting, respectively) and OASIS-1 trials (omadacycline, 12.4% and 5.3%; linezolid, 9.9% and 5.0% for nausea and vomiting, respectively) [58]. Discontinuation rates were 5.5% of 382 patients with CABP (7.0% for comparator) and 1.7% of 691 patients with ABSSSI (1.5% for comparator) [58,64]. An imbalance of mortality was observed for omadacycline (2%) versus moxifloxacin (1%) in the CABP OPTIC study, although the cause of this imbalance has not been established.

### Special patient populations

Use of tetracycline-class drugs in pregnancy and for children under 8 years of age should be avoided, unless the benefit outweighs the risk, due to the risk of permanent tooth discolouration, enamel hypoplasia, and inhibition of bone growth. No dosage adjustments are needed for any of the third-generation tetracyclines in patients with renal impairment or mild-to-moderate hepatic impairment. However, dosage adjustments are required for eravacycline and tigecycline in patients with severe hepatic impairment (Child–Pugh Class C). Patient weight can impact PK and outcomes for some antibiotics, which may therefore require patient-specific dosing. Studies in most third-generation tetracycline-class antibiotics indicate that no adjustments are needed based on body weight [84], although dosing of eravacycline is based on 1 mg/kg body weight [12]. Efficacy of omadacycline and eravacycline is consistent across body mass index (BMI) groupings [57,85,86], and the PK of tigecycline is similar in patients with class III obesity (BMI ≥40 kg/m^2^) or healthy weight [87]. Additionally, safety data indicate tolerance of higher-dose eravacycline in patients with increased BMI [85].

Drug-drug interactions (DDIs) leading to adverse drug events are a common cause of emergency department visit and/or hospitalisation [88,89]. Patients, particularly at risk of these DDIs, are older patients who are on many long-standing medications, immunocompromised patients, and others with multiple medical problems [90]. Based on their PK/PD characteristics and ability to be used as monotherapy, newer tetracycline-class agents can be used to minimise the risk of severe drug-drug interactions in these high-risk patients.

Patients with labeled drug-drug penicillin allergy in the past have had multiple negative outcomes associated with the use of alternative antimicrobial agents including the risk of antimicrobial treatment failure, antimicrobial resistance, adverse drug reactions from the use of a broader-spectrum or alternative antibiotic, increased risk of CDI, and increased healthcare costs [91,92]. In these patients, a newer generation of tetracycline-class agents could provide a therapeutic option that is not only efficacious but also mitigates the risk of various negative sequelae.

The risk of recurrent CDI is higher in patients aged ≥65 years, or who have compromised immunity, prior severe CDI, or ribotype 027/078/244 infections [93]. If these patients require ongoing or repeat antibiotic therapy immediately after the initial episode of CDI, then antibiotics with known increased risk of CDI (clindamycin, fluoroquinolones, β-lactam agents) could be avoided.

### Re-engaging with tetracycline-class drugs – where could patients benefit?

Antimicrobial stewardship involves using the right antibiotic agent, at the right time, for the right patient. While the clinical utility of first- and second-generation tetracycline-class drugs has been decreasing, third-generation drugs overcome the most common acquired tetracycline resistance mechanisms and therefore may play an important role in antimicrobial stewardship and in reducing “collateral damage” of antibiotic therapies. The increases in extended-spectrum β-lactamase (ESBL)-producing bacteria have limited the use of β-lactams in both hospital and outpatient settings [94,95]. The broad-spectrum activity of the third-generation tetracycline-class agents, combined with their activity being unaffected by common resistance genes, including β-lactamases, makes them suitable for treating a wide range of infections including those caused by drug-resistant strains, such as MRSA, vancomycin-resistant enterococci, tetracycline-resistant *S. pneumoniae*, and anaerobic pathogens [35,96–101]. Additionally, these drugs can be used as a monotherapy to treat polymicrobial infections, such as IAIs and skin structure infections, thereby minimising the impact on global antimicrobial resistance. The emerging threat of the *tet*(X) which causes resistance to all tetracyclines but not to other antibiotic classes, warrants continued monitoring of tetracycline resistance, further restriction of tetracyclines (and other antibiotics) in farm animals, and robust antimicrobial stewardship to promote their optimal use including considerations to reduce selective pressure potential by deprioritizing the use of tetracyclines for pathogens that may harbour *tet*(X). In addition to the currently approved indications for the third-generation tetracycline-class drugs, clinical trials are ongoing in diabetes-related infections (NCT04144374), cystic fibrosis (NCT04460586), pulmonary *Mycobacterium abscessus* complex (NCT04922554) and multidrug-resistant blood-stream infections (NCT04489459, NCT04876430).

The reduced risk of CDI compared with other antibiotic classes such as β-lactams and fluoroquinolones suggest that third-generation tetracycline-class drugs may present alternative antibiotic treatment options for patients with an increased risk of primary or recurrent CDI.

As third-generation tetracycline-class drugs do not require dose adjustments for end-stage renal impairment or mild-to-moderate hepatic impairment and have a limited number of DDIs, they provide a suitable therapy option for patients who otherwise could experience reduced efficacy with other antimicrobial agents, and for older adults who are often taking multiple chronic medications.

Finally, the mode of delivery and frequency of administration may provide benefits to patients over previous therapies. For example, the availability of doxycycline, minocycline, and omadacycline as IV-to-oral therapies could potentially reduce inpatient treatment times, and oral-only options allow outpatient treatment of patients with appropriate indications who may otherwise have required IV therapy. Similarly, oral medications including antibiotics that require infrequent dosing can also improve treatment adherence and patient compliance [102].

In summary, in an era of multidrug antibiotic resistance, additional treatment options are needed to address infections in complex patient populations. The latest generation of tetracycline-class antibiotics overcome the most common mechanisms of tetracycline resistance that limit the use of prior tetracyclines and are well suited to treat various infectious syndromes including skin-soft tissue infections, community acquired pneumonia and intra-abdominal infections. While all antibiotics are subject to the development of resistance, judicious use of tetracyclines not only provides another class of antibiotic activity but also helps by not promoting resistance to other commonly used antibiotic classes (e.g. beta-lactams, fluoroquinolones). Therefore, the third-generation tetracycline class has the potential to play a key role in the treatment of a broad range of bacterial infections to provide a safe and effective treatment option for patients and to support antibiotic stewardship initiatives.

## Data Availability

Data sharing is not applicable to this article as no new data were created or analysed in this study.

## References

[1] Centers for Disease Control and Prevention. Antibiotic resistant threats in the United States; 2019. https://www.cdc.gov/drugresistance/biggest-threats.html.

[2] Nelson RE, Hatfield KM, Wolford H, et al. National estimates of healthcare costs associated with multidrug-resistant bacterial infections among hospitalized patients in the United States. Clin Infect Dis. 2021;72(Suppl 1):S17–S26. 33512523 10.1093/cid/ciaa1581 PMC11864165

[3] The 10 x '20 initiative: pursuing a global commitment to develop 10 new antibacterial drugs by 2020. Clin Infect Dis. 2010;50(8):1686–1083. 10.1086/652237 20214473

[4] Talbot GH, Jezek A, Murray BE, et al. The Infectious Diseases Society of America's 10 × '20 Initiative (10 New Systemic Antibacterial Agents US Food and Drug Administration Approved by 2020): is 20 × '20 a possibility? Clin Infect Dis. 2019;69(1):1–11. 30715222 10.1093/cid/ciz089

[5] Chopra I, Roberts M. Tetracycline antibiotics: mode of action, applications, molecular biology, and epidemiology of bacterial resistance. Microbiol Mol Biol Rev. 2001;65(2):232–260. 11381101 10.1128/MMBR.65.2.232-260.2001 PMC99026

[6] Chukwudi CU. rRNA binding sites and the molecular mechanism of action of the tetracyclines. Antimicrob Agents Chemother. 2016;60(8):4433–4441. 27246781 10.1128/AAC.00594-16 PMC4958212

[7] Eliopoulos GM, Eliopoulos GM, Roberts MC. Tetracycline therapy: update. Clin Infect Dis. 2003;36(4):462–467. 12567304 10.1086/367622

[8] Pharma AA. Tetracycline (tetracycline hydrochloride) prescribing information. 2010. https://www.aapharma.ca/downloads/en/PIL/Tetracycline_PI.pdf.

[9] Paratek Pharmaceuticals. Nuyzra(R) (omadacycline) prescribing information. 2020. https://www.nuzyra.com/nuzyra-pi.pdf.

[10] Pfizer. TYGACIL® (tigecycline) prescribing information. 2005. https://www.pfizermedicalinformation.com/en-us/tygacil.

[11] Apotex Inc. Doxycycline hyclate prescribing information. 2016. https://pdf.hres.ca/dpd_pm/00037247.PDF.

[12] Tetraphase Pharmaceuticals. XERAVA (eravacycline) prescribing information. 2020. https://www.tphase.com/products/xerava/.

[13] Valeant Pharmaceuticals. MINOCIN® (minocycline hydrochloride) prescribing information. 2019. https://www.fda.gov/media/121327/download.

[14] Dowling HF, Lepper MH, Jackson GG. Observations on the epidemiological spread of antibiotic-resistant staphylococci, with measurements of the changes in sensitivity to penicillin and aureomycin. Am J Public Health Nations Health. 1953;43(7):860–868. 13065540 10.2105/ajph.43.7.860 PMC1620328

[15] Lowbury EJ, Cason JS. Aureomycin and erythromycin therapy for str. pyogenes in burns. Br Med J. 1954;2(4893):914–915. 13199343 10.1136/bmj.2.4893.914 PMC2079339

[16] Percival A. Increased incidence of tetracycline-resistant pneumococci in Liverpool in 1968. Lancet. 1969;1(7603):998–1000. 4181183 10.1016/s0140-6736(69)91799-1

[17] Emmerson AM, Jones AM. The quinolones: decades of development and use. J Antimicrob Chemother. 2003;51(90001):13–20. 10.1093/jac/dkg208 12702699

[18] Markley JL, Wencewicz TA. Tetracycline-inactivating enzymes. Front Microbiol. 2018;9:1058. 29899733 10.3389/fmicb.2018.01058 PMC5988894

[19] Grossman TH. Tetracycline antibiotics and resistance. Cold Spring Harb Perspect Med. 2016;6(4):a025387. 26989065 10.1101/cshperspect.a025387 PMC4817740

[20] Fang LX, Chen C, Cui C-Y, et al. Emerging high-level tigecycline resistance: novel tetracycline destructases spread via the mobile tet(X). BioEssays. 2020;42(8):2000014. 10.1002/bies.202000014 32567703

[21] Kadavy DR, Hornby JM, Haverkost T, et al. Natural antibiotic resistance of bacteria isolated from larvae of the oil fly, *Helaeomyia petrolei*. Appl Environ Microbiol. 2000;66(11):4615–4619. 11055901 10.1128/aem.66.11.4615-4619.2000 PMC92357

[22] Stock I. Natural antibiotic susceptibility of *Proteus* spp., with special reference to *P. mirabilis* and *P. penneri* strains. J Chemother. 2003;15(1):12–26. 12678409 10.1179/joc.2003.15.1.12

[23] Li XZ, Livermore DM, Nikaido H. Role of efflux pump(s) in intrinsic resistance of *Pseudomonas aeruginosa*: resistance to tetracycline, chloramphenicol, and norfloxacin. Antimicrob Agents Chemother. 1994;38(8):1732–1741. 7986003 10.1128/aac.38.8.1732 PMC284630

[24] Diekema DJ, Pfaller MA, Shortridge D, et al. Twenty-year trends in antimicrobial susceptibilities among *Staphylococcus aureus* from the SENTRY antimicrobial surveillance program. Open Forum Infect Dis. 2019;6(Suppl 1):S47–s53. 30895214 10.1093/ofid/ofy270 PMC6419894

[25] Gupta V, Yu KC, Schranz J, et al. A multicenter evaluation of the US prevalence and regional variation in macrolide-resistant *S. pneumoniae* in ambulatory and hospitalized adult patients in the United States. Open Forum Infect Dis. 2021;8(7):ofab063. 34250183 10.1093/ofid/ofab063 PMC8266646

[26] Huband MD, Pfaller MA, Streit JM, et al. Activity of omadacycline against 7,000 bacterial isolates from the United States in the SENTRY Antimicrobial Surveillance Program (2020). World Microbe Forum 2021. June 20–24.

[27] Jones RN, Stilwell MG, Wilson ML, et al. Contemporary tetracycline susceptibility testing: doxycycline MIC methods and interpretive criteria (CLSI and EUCAST) performance when testing gram-positive pathogens. Diagn Microbiol Infect Dis. 2013;76(1):69–72. 23490012 10.1016/j.diagmicrobio.2013.01.023

[28] Jones RN, Wilson ML, Weinstein MP, et al. Contemporary potencies of minocycline and tetracycline HCL tested against gram-positive pathogens: SENTRY program results using CLSI and EUCAST breakpoint criteria. Diagn Microbiol Infect Dis. 2013;75(4):402–405. 23514756 10.1016/j.diagmicrobio.2013.01.022

[29] Nguyen F, Starosta AL, Arenz S, et al. Tetracycline antibiotics and resistance mechanisms. Biol Chem. 2014;395(5):559–575. 24497223 10.1515/hsz-2013-0292

[30] Petersen PJ, Jacobus NV, Weiss WJ, et al. In vitro and in vivo antibacterial activities of a novel glycylcycline, the 9-t-butylglycylamido derivative of minocycline (GAR-936). Antimicrob Agents Chemother. 1999;43(4):738–744. 10103174 10.1128/aac.43.4.738 PMC89200

[31] Schwartz BS, Graber CJ, Diep BA, et al. Doxycycline, not minocycline, induces its own resistance in multidrug-resistant, community-associated methicillin-resistant *Staphylococcus aureus* clone USA300. Clin Infect Dis. 2009;48(10):1483–1484. 19374563 10.1086/598510

[32] Mendes RE, Huband MD, Streit JM, et al. Omadacycline invitro activity against a molecularly characterized collection of clinical isolates with known acquired tetracycline resistance mechanisms. Diagn Microbiol Infect Dis. 2020;97(3):115054. 32376058 10.1016/j.diagmicrobio.2020.115054

[33] Kowalska-Krochmal B, Dudek-Wicher R. The minimum inhibitory concentration of antibiotics: methods, interpretation, clinical relevance. Pathogens. 2021;10(2):165. 33557078 10.3390/pathogens10020165 PMC7913839

[34] Macone AB, Caruso BK, Leahy RG, et al. In vitro and in vivo antibacterial activities of omadacycline, a novel aminomethylcycline. Antimicrob Agents Chemother. 2014;58(2):1127–1135. 24295985 10.1128/AAC.01242-13 PMC3910882

[35] Sutcliffe JA, O'Brien W, Fyfe C, et al. Antibacterial activity of eravacycline (TP-434), a novel fluorocycline, against hospital and community pathogens. Antimicrob Agents Chemother. 2013;57(11):5548–5558. 23979750 10.1128/AAC.01288-13 PMC3811277

[36] Lijfrock V, Morgan S, Hwang S, et al. 1613. Global 2018 surveillance of eravacycline against gram-negative pathogens, including multi-drug resistant isolates. Open Forum Infect Dis. 2020;7(Supplement_1):S800–S.

[37] Morgan S, Hwang S, Efimova E, et al. 915. Global 2018 surveillance of eravacycline against gram-positive pathogens, including resistant isolates. Open Forum Infect Dis. 2020;7(Supplement_1):S492–S.

[38] Linkevicius M, Sandegren L, Andersson DI. Potential of tetracycline resistance proteins to evolve tigecycline resistance. Antimicrob Agents Chemother. 2016;60(2):789–796. 26596936 10.1128/AAC.02465-15 PMC4750697

[39] Sun Y, Cai Y, Liu X, et al. The emergence of clinical resistance to tigecycline. Int J Antimicrob Agents. 2013;41(2):110–116. 23127485 10.1016/j.ijantimicag.2012.09.005

[40] Wen Z, Shang Y, Xu G, et al. Mechanism of eravacycline resistance in clinical *Enterococcus faecalis* isolates from China. Front Microbiol. 2020;11(916):916. 32523563 10.3389/fmicb.2020.00916 PMC7261854

[41] He T, Wang R, Liu D, et al. Emergence of plasmid-mediated high-level tigecycline resistance genes in animals and humans. Nat Microbiol. 2019;4(9):1450–1456. 31133751 10.1038/s41564-019-0445-2

[42] Sun J, Chen C, Cui CY, et al. Plasmid-encoded tet(X) genes that confer high-level tigecycline resistance in *Escherichia coli*. Nat Microbiol. 2019;4(9):1457–1464. 31235960 10.1038/s41564-019-0496-4 PMC6707864

[43] Hoban DJ, Reinert RR, Bouchillon SK, et al. Global in vitro activity of tigecycline and comparator agents: Tigecycline evaluation and surveillance trial 2004-2013. Ann Clin Microbiol Antimicrob. 2015;14:27. 25958201 10.1186/s12941-015-0085-1 PMC4489028

[44] Muralidharan G, Micalizzi M, Speth J, et al. Pharmacokinetics of tigecycline after single and multiple doses in healthy subjects. Antimicrob Agents Chemother. 2005;49(1):220–229. 15616299 10.1128/AAC.49.1.220-229.2005 PMC538906

[45] Center for Drug Evaluation and Research. Eravacycline multi-discipline review; 2018. https://www.accessdata.fda.gov/drugsatfda_docs/nda/2018/211109Orig1s000MultidisciplineR.pdf.

[46] Zhao M, Lepak AJ, Marchillo K, et al. In vivo pharmacodynamic target assessment of eravacycline against *Escherichia coli* in a murine thigh infection model. Antimicrob Agents Chemother. 2017;61(7):e00250–17. 28416552 10.1128/AAC.00250-17 PMC5487610

[47] Agwuh KN, MacGowan A. Pharmacokinetics and pharmacodynamics of the tetracyclines including glycylcyclines. J Antimicrob Chemother. 2006;58(2):256–265. 16816396 10.1093/jac/dkl224

[48] Burgos RM, Rodvold KA. Omadacycline: a novel aminomethylcycline. Infect Drug Resist. 2019;12:1895–1915. 31308710 10.2147/IDR.S171352 PMC6613460

[49] Cunha BA, Domenico P, Cunha CB. Pharmacodynamics of doxycycline. Clin Microbiol Infect. 2000;6(5):270–273. 11168126 10.1046/j.1469-0691.2000.00058-2.x

[50] Tanaka SK, Steenbergen J, Villano S. Discovery, pharmacology, and clinical profile of omadacycline, a novel aminomethylcycline antibiotic. Bioorg Med Chem. 2016;24(24):6409–6419. 27469981 10.1016/j.bmc.2016.07.029

[51] Center for Drug Evaluation and Research. Xerava (eravacycline): NDA multidisciplinary review and evaluation 2017. https://www.accessdata.fda.gov/drugsatfda_docs/nda/2018/211109Orig1s000MultidisciplineR.pdf.

[52] LaPlante KL, Leonard SN, Andes DR, et al. Activities of clindamycin, daptomycin, doxycycline, linezolid, trimethoprim-sulfamethoxazole, and vancomycin against community-associated methicillin-resistant *Staphylococcus aureus* with inducible clindamycin resistance in murine thigh infection and in vitro pharmacodynamic models. Antimicrob Agents Chemother. 2008;52(6):2156–2162. 18411321 10.1128/AAC.01046-07 PMC2415789

[53] Rodvold KA, Pai MP. Pharmacokinetics and pharmacodynamics of oral and intravenous omadacycline. Clin Infect Dis. 2019;69(Suppl 1):S16–s22. 31367744 10.1093/cid/ciz309 PMC6669312

[54] Lepak AJ, Zhao M, Marchillo K, et al. In vivo pharmacodynamics of omadacycline against *Staphylococcus aureus* in the neutropenic murine thigh infection model. Antimicrob Agents Chemother. 2019;63(7):e00634–19. 31036691 10.1128/AAC.00624-19 PMC6591633

[55] Lepak AJ, Zhao M, Marchillo K, et al. In vivo pharmacodynamic evaluation of omadacycline (PTK 0796) against *Streptococcus pneumoniae* in the murine pneumonia model. Antimicrob Agents Chemother. 2017;61(5):e02368–16. 28193651 10.1128/AAC.02368-16 PMC5404567

[56] Crandon JL, Banevicius MA, Nicolau DP. Pharmacodynamics of tigecycline against phenotypically diverse *Staphylococcus aureus* isolates in a murine thigh model. Antimicrob Agents Chemother. 2009;53(3):1165–1169. 19114676 10.1128/AAC.00647-08 PMC2650523

[57] Pai MP, Wilcox MH, Chitra S, et al. Safety and efficacy of omadacycline by BMI categories and diabetes history in two phase III randomized studies of patients with acute bacterial skin and skin structure infections. J Antimicrob Chemother. 2021;76(5):1315–1322. 33458763 10.1093/jac/dkaa558 PMC8050767

[58] Stets R, Popescu M, Gonong JR, et al. Omadacycline for community-acquired bacterial pneumonia. N Engl J Med. 2019;380(6):517–527. 30726692 10.1056/NEJMoa1800201

[59] Bergallo C, Jasovich A, Teglia O, et al. Safety and efficacy of intravenous tigecycline in treatment of community-acquired pneumonia: results from a double-blind randomized phase 3 comparison study with levofloxacin. Diagn Microbiol Infect Dis. 2009;63(1):52–61. 18990531 10.1016/j.diagmicrobio.2008.09.001

[60] Tanaseanu C, Milutinovic S, Calistru PI, et al. Efficacy and safety of tigecycline versus levofloxacin for community-acquired pneumonia. BMC Pulm Med. 2009;9:44. 19740418 10.1186/1471-2466-9-44 PMC2753558

[61] US Department of Health and Human Services FaDA, Center for Drug Evaluation and Research (CDER). Guidance for Industry. Community-acquired bacterial pneumonia: developing drugs for treatment; 2014. http://www.fda.gov/media/75149/download.

[62] US Department of Health and Human Services FaDA, Center for Drug Evaluation and Research (CDER). Guidance for Industry. Acute bacterial skin and skin structure infections: developing drugs for treatment; 2013. http://www.fda.gov/media/71052/download.

[63] O'Riordan W, Cardenas C, Shin E, et al. Once-daily oral omadacycline versus twice-daily oral linezolid for acute bacterial skin and skin structure infections (OASIS-2): a phase 3, double-blind, multicentre, randomised, controlled, non-inferiority trial. Lancet Infect Dis. 2019;19(10):1080–1090. 31474458 10.1016/S1473-3099(19)30275-0

[64] O'Riordan W, Green S, Overcash JS, et al. Omadacycline for acute bacterial skin and skin-structure infections. N Engl J Med. 2019;380(6):528–538. 30726689 10.1056/NEJMoa1800170

[65] Breedt J, Teras J, Gardovskis J, et al. Safety and efficacy of tigecycline in treatment of skin and skin structure infections: results of a double-blind phase 3 comparison study with vancomycin-aztreonam. Antimicrob Agents Chemother. 2005;49(11):4658–4666. 16251309 10.1128/AAC.49.11.4658-4666.2005 PMC1280174

[66] Sacchidanand S, Penn RL, Embil JM, et al. Efficacy and safety of tigecycline monotherapy compared with vancomycin plus aztreonam in patients with complicated skin and skin structure infections: results from a phase 3, randomized, double-blind trial. Int J Infect Dis. 2005;9(5):251–261. 16099700 10.1016/j.ijid.2005.05.003

[67] Solomkin J, Evans D, Slepavicius A, et al. Assessing the efficacy and safety of eravacycline vs ertapenem in complicated intra-abdominal infections in the investigating Gram-Negative Infections Treated with Eravacycline (IGNITE 1) trial: a randomized clinical trial. JAMA Surg. 2017;152(3):224–232. 27851857 10.1001/jamasurg.2016.4237

[68] Solomkin JS, Gardovskis J, Lawrence K, et al. IGNITE4: Results of a phase 3, randomized, multicenter, prospective trial of eravacycline vs meropenem in the treatment of complicated intraabdominal infections. Clin Infect Dis. 2019;69(6):921–929. 30561562 10.1093/cid/ciy1029 PMC6735687

[69] Fomin P, Beuran M, Gradauskas A, et al. Tigecycline is efficacious in the treatment of complicated intra-abdominal infections. Int J Surg. 2005;3(1):35–47. 17462257 10.1016/j.ijsu.2005.03.011

[70] Oliva ME, Rekha A, Yellin A, et al. A multicenter trial of the efficacy and safety of tigecycline versus imipenem/cilastatin in patients with complicated intra-abdominal infections [Study ID Numbers: 3074A1-301-WW; ClinicalTrials.gov Identifier: NCT00081744]. BMC Infect Dis. 2005;5:88. 16236177 10.1186/1471-2334-5-88 PMC1277826

[71] Bjellerup M, Ljunggren B. Photohemolytic potency of tetracyclines. J Invest Dermatol. 1985;84(4):262–264. 3981037 10.1111/1523-1747.ep12265336

[72] Brown KA, Khanafer N, Daneman N, et al. Meta-analysis of antibiotics and the risk of community-associated *Clostridium difficile* infection. Antimicrob Agents Chemother. 2013;57(5):2326–2332. 23478961 10.1128/AAC.02176-12 PMC3632900

[73] Slimings C, Riley TV. Antibiotics and hospital-acquired *Clostridium difficile* infection: update of systematic review and meta-analysis. J Antimicrob Chemother. 2014;69(4):881–891. 24324224 10.1093/jac/dkt477

[74] Deshpande A, Pasupuleti V, Thota P, et al. Community-associated *Clostridium difficile* infection and antibiotics: a meta-analysis. J Antimicrob Chemother. 2013;68(9):1951–1961. 23620467 10.1093/jac/dkt129

[75] Tariq R, Cho J, Kapoor S, et al. Low risk of primary *Clostridium difficile* infection with tetracyclines: a systematic review and meta analysis. Clin Infect Dis. 2018;66(4):514–522. 29401273 10.1093/cid/cix833

[76] Kechagias KS, Chorepsima S, Triarides NA, et al. Tigecycline for the treatment of patients with *Clostridium difficile* infection: an update of the clinical evidence. Eur J Clin Microbiol Infect Dis. 2020;39(6):1053–1058. 31927652 10.1007/s10096-019-03756-z

[77] Opal S, File TM Jr, van der Poll T, et al. An integrated safety summary of omadacycline, a novel aminomethylcycline antibiotic. Clin Infect Dis. 2019;69(Suppl 1):S40–S7. 31367740 10.1093/cid/ciz398 PMC6669290

[78] Van Hise N, Petrak RM, Skorodin NC, et al. A real-world assessment of clinical outcomes and safety of eravacycline: a novel fluorocycline. Infect Dis Ther. 2020;9(4):1017–1028. 33063176 10.1007/s40121-020-00351-0 PMC7680490

[79] Moura IB, Buckley AM, Ewin D, et al. Omadacycline gut microbiome exposure does not induce clostridium difficile proliferation or toxin production in a model that simulates the proximal, medial, and distal human Colon. Antimicrob Agents Chemother. 2019;63(2):e01581–18. 30455242 10.1128/AAC.01581-18 PMC6355569

[80] Garey KW, Rose W, Gunter K, et al. Omadacycline and Clostridioides difficile: a systematic review of preclinical and clinical evidence. Ann Med. 10.1177/10600280221089007 PMC9874691 35656828

[81] Lauf L, Ozsvár Z, Mitha I, et al. Phase 3 study comparing tigecycline and ertapenem in patients with diabetic foot infections with and without osteomyelitis. Diagn Microbiol Infect Dis. 2014;78(4):469–480. 24439136 10.1016/j.diagmicrobio.2013.12.007

[82] Wallace RJ Jr, Dukart G, Brown-Elliott BA, et al. Clinical experience in 52 patients with tigecycline-containing regimens for salvage treatment of *Mycobacterium abscessus* and *Mycobacterium chelonae* infections. J Antimicrob Chemother. 2014;69(7):1945–1953. 24633206 10.1093/jac/dku062 PMC4054987

[83] Solomkin JS, Ramesh MK, Cesnauskas G, et al. Phase 2, randomized, double-blind study of the efficacy and safety of two dose regimens of eravacycline versus ertapenem for adult community-acquired complicated intra-abdominal infections. Antimicrob Agents Chemother. 2014;58(4):1847–1854. 24342651 10.1128/AAC.01614-13 PMC4023720

[84] Pai MP. Antimicrobial dosing in specific populations and novel clinical methodologies: obesity. Clin Pharmacol Ther. 2021;109(4):942–951. 33523485 10.1002/cpt.2181 PMC8855475

[85] Asempa TE, Izmailyan S, Lawrence K, et al. Efficacy and safety of eravacycline in obese patients: a post hoc analysis of pooled data from the IGNITE1 and IGNITE4 clinical trials. Open Forum Infect Dis. 2020;7(12):ofaa548. 33365356 10.1093/ofid/ofaa548 PMC7747372

[86] Pai MP, Wilcox M, Chitra S, et al. Safety and efficacy of omadacycline by body mass index in patients with community-acquired bacterial pneumonia: subanalysis from a randomized controlled trial. Respir Med. 2021;184:106442. 34058682 10.1016/j.rmed.2021.106442

[87] Pai MP. Serum and urine pharmacokinetics of tigecycline in obese class III and normal weight adults. J Antimicrob Chemother. 2014;69(1):190–199. 23883872 10.1093/jac/dkt299

[88] Swart F, Bianchi G, Lenzi J, et al. Risk of hospitalization from drug-drug interactions in the elderly: real-world evidence in a large administrative database. Aging. 2020;12(19):19711–19739. 33024058 10.18632/aging.104018 PMC7732312

[89] Dookeeram D, Bidaisee S, Paul JF, et al. Polypharmacy and potential drug-drug interactions in emergency department patients in the Caribbean. Int J Clin Pharm. 2017;39(5):1119–1127. 28795285 10.1007/s11096-017-0520-9 PMC5686268

[90] Bjerrum L, Gonzalez Lopez-Valcarcel B, Petersen G. Risk factors for potential drug interactions in general practice. Eur J Gen Pract. 2008;14(1):23–29. 18464169 10.1080/13814780701815116

[91] MacFadden DR, LaDelfa A, Leen J, et al. Impact of reported beta-lactam allergy on inpatient outcomes: a multicenter prospective cohort study. Clin Infect Dis. 2016;63(7):904–910. 27402820 10.1093/cid/ciw462

[92] Assimon MM, Pun PH, Wang L, et al. Analysis of respiratory fluoroquinolones and the risk of sudden cardiac death among patients receiving hemodialysis. JAMA Cardiol. 2021;7(1):75. 10.1001/jamacardio.2021.4234 PMC8756330 34668928

[93] Kelly CP. Can we identify patients at high risk of recurrent *Clostridium difficile* infection? Clin Microbiol Infect. 2012;18:21–27. 10.1111/1469-0691.12046 23121551

[94] Thaden JT, Fowler VG, Sexton DJ, et al. Increasing incidence of extended-spectrum β-lactamase-producing *Escherichia coli* in community hospitals throughout the southeastern United States. Infect Control Hosp Epidemiol. 2016;37(1):49–54. 26458226 10.1017/ice.2015.239 PMC4748740

[95] Coque T, Baquero F, Canton R. Increasing prevalence of ESBL-producing *Enterobacteriaceae* in Europe. Euro Surveill. 2008;13(47):19044. 19021958

[96] Stone TJ, Kilic A, Williamson J, et al. 1267. Comparative activity of omadacycline against extended-spectrum beta-lactamase positive and negative *Escherichia coli* and *Klebsiella pneumoniae* strains recovered from urine specimens. Open Forum Infect Dis. 2020;7(Supplement_1):S650–S650.

[97] Sader HS, Flamm RK, Jones RN. Tigecycline activity tested against antimicrobial resistant surveillance subsets of clinical bacteria collected worldwide (2011). Diagn Microbiol Infect Dis. 2013;76(2):217–221. 23522845 10.1016/j.diagmicrobio.2013.02.009

[98] Zhanel GG, Cheung D, Adam H, et al. Review of eravacycline, a novel fluorocycline antibacterial agent. Drugs. 2016;76(5):567–588. 26863149 10.1007/s40265-016-0545-8

[99] Zhanel GG, Esquivel J, Zelenitsky S, et al. Omadacycline: a novel oral and intravenous aminomethylcycline antibiotic agent. Drugs. 2020;80(3):285–313. 31970713 10.1007/s40265-020-01257-4

[100] Xiao M, Huang J-J, Zhang G, et al. Antimicrobial activity of omadacycline in vitro against bacteria isolated from 2014 to 2017 in China, a multi-center study. BMC Microbiol. 2020;20(1):350. 33198626 10.1186/s12866-020-02019-8 PMC7667747

[101] Karlowsky JA, Steenbergen J, Zhanel GG. Microbiology and preclinical review of omadacycline. Clin Infect Dis. 2019;69(Suppl 1):S6–S15. 31367743 10.1093/cid/ciz395 PMC6669291

[102] Srivastava K, Arora A, Kataria A, et al. Impact of reducing dosing frequency on adherence to oral therapies: a literature review and meta-analysis. Patient Prefer Adherence. 2013;7:419–434. 23737662 10.2147/PPA.S44646 PMC3669002

[103] Pfaller MA, Huband MD, Shortridge D, et al. Surveillance of omadacycline activity tested against clinical isolates from the United States and Europe: report from the SENTRY antimicrobial surveillance program, 2016 to 2018. Antimicrob Agents Chemother. 2020;64(5):e02488–19. 32071045 10.1128/AAC.02488-19 PMC7179604

[104] Morrissey I, Hawser S, Lob SH, et al. In vitro activity of eravacycline against gram-positive bacteria isolated in clinical laboratories worldwide from 2013 to 2017. Antimicrob Agents Chemother. 2020;64(3):e01715–19. 31843997 10.1128/AAC.01715-19 PMC7038300

[105] Grossman TH, Murphy TM, Slee AM, et al. Eravacycline (TP-434) is efficacious in animal models of infection. Antimicrob Agents Chemother. 2015;59(5):2567–2571. 25691636 10.1128/AAC.04354-14 PMC4394802

[106] Hawkey PM. The origins and molecular basis of antibiotic resistance. BMJ 1998;317:657–660. 9727999 10.1136/bmj.317.7159.657 PMC1113838

